# Harmonization of Longitudinal Diffusion Tensor Imaging Data of the Pediatric Cervical and Thoracic Spinal Cord at 3T Using Longitudinal ComBat

**DOI:** 10.21203/rs.3.rs-4536023/v1

**Published:** 2024-07-01

**Authors:** Yutong Li, Devon M Middleton, Andrew Chen, Russell T Shinohara, Laura Krisa, Scott H Faro, Mary Jane Mulcahey, Feroze B Mohamed

**Affiliations:** Thomas Jefferson University, Sidney Kimmel Medical College; Thomas Jefferson University; University of Pennsylvania Perelman School of Medicine; University of Pennsylvania Perelman School of Medicine; Thomas Jefferson University; Thomas Jefferson University; Thomas Jefferson University; Thomas Jefferson University

**Keywords:** Harmonization, Scanner effects, Diffusion tensor imaging, spinal cord injury, Longitudinal ComBat, Longitudinal scans

## Abstract

Diffusion tensor imaging (DTI) of the spinal cord has been extensively used to identify biomarkers for spinal cord pathology. Previously, the longitudinal ComBat (longComBat) technique was examined to reduce scanner effects in multi-site, multi-scanner spinal cord DTI data. This study aimed to assess its effectiveness on longitudinal scans using a single-scanner pediatric dataset, including healthy and spinal cord injury (SCI) subjects. Two identical datasets were collected from 42 healthy and 27 SCI subjects with a 2-hour interval between scans on a 3T Siemens MRI scanner. Axial DTI images of the entire cervical and thoracic spinal cord were obtained, and various average diffusion tensor metrics (FA, MD, RD, & AD) were measured at each vertebral level. Pearson correlation and intraclass correlation coefficients were used to evaluate inter- and intra-subject agreement pre- and post-harmonization. Minimal improvement in agreement was observed with the mean square residual (MSR) model, while the restricted maximum likelihood estimator (REML) model showed reduced intra-subject agreement in all the tensor metrics. The significant variability between longitudinal DTI scans within a single scanner was likely due to physiological motion rather than scanner effects. Post-harmonization using the longComBat MSR model showed limited improvement in agreement.

## Introduction

Diffusion tensor imaging (DTI) has emerged as a powerful modality for examining microstructural alterations within the spinal cord, offering invaluable insights into various neurological conditions such as traumatic and non-traumatic spinal cord injury, degenerative diseases, and tumors [1–3]. DTI parameters including fractional anisotropy (FA), mean diffusivity (MD), axial diffusivity (AD), radial diffusivity (RD), apparent diffusion coefficient (ADC), and relative anisotropy (RA) have been demonstrated to be good indicators of normative white matter microstructure and potential predictors of demyelination in pathological states in human and animal studies [3, 9–13].

Numerous studies have investigated adult [2, 3] and pediatric [4–8] spinal cord DTI data.

DTI metrics have shown to be potential biomarkers for injury and disease, but the quantitative interpretation of DTI can be challenging, especially for the spinal cord. The unique architecture of the spinal cord, characterized by its small size, complex fiber organization, and susceptibility to physiological motion, poses significant challenges for accurate imaging and interpretation of DTI data. Furthermore, the lack of standardized acquisition protocols and the inherent variability across magnetic resonance (MR) scanners further complicate the comparison and synthesis of findings across studies and clinical sites. Different MR scanners vary based on magnetic field strength, gradient performance, pulse sequence designs, processing techniques and calculation methods [14]. In recent years, efforts have been made to address these challenges through the development of advanced imaging techniques. Reduced field-of-view (rFoV) diffusion-weighted imaging sequences have demonstrated promise in reducing geometric distortions and artifacts, particularly beneficial for the small dimensions of the spinal cord [5, 15–19]. Despite these advancements, significant gaps remain in our understanding of the reproducibility and reliability of DTI measurements in the spinal cord [4].

The clinical translation of DTI biomarkers for spinal cord pathologies relies heavily on the establishment of robust and standardized imaging protocols, as well as validated harmonization techniques to ensure consistency and comparability across diverse patient populations and clinical settings. Longitudinal ComBat (longComBat), an empirical Bayesian method, is one of a harmonization method that removes additive scanner effects and corrects multiplicative scanner effects by removing heteroscedasticity of model errors across scanners [20]. It is a generalization of a method was originally used in genomics, which has been adapted for brain functional MRI and DTI with promising results [20]. This technique has never been applied to spinal cord imaging prior to our studies. In our prior study, we demonstrated the efficacy of longComBat in decreasing scanner effects on the data from different scanners and field strengths [14].

Few studies have been conducted to show spinal cord DTI reproducibility within scanner [4]. In this study, we concentrate on demonstrating the efficacy of longComBat in decreasing scan-rescan variability on the longitudinal data obtained from the same scanner of the cervical and thoracic spinal cord. We examined the variability of DTI of the spinal cord between longitudinal scans with a single 3T Siemens MR scanner by scanning a sample of forty-two healthy pediatric subjects and twenty-seven pediatric subjects before and after a 2-hour interval. We continue to show that harmonization of human spinal cord DTI data is a crucial prerequisite for facilitating longitudinal and multisite clinical research as well as clinical trials.

## Results

### Attrition

Thirty-six out of forty-two healthy subjects successfully completed the entire protocol, while twenty-six out of twenty-seven subjects with spinal cord injury completed the full protocol. Among those who completed the full protocol, thirty-two healthy subjects and thirteen spinal cord injury subjects had complete DTI metrics data for the entire spinal cord, allowing for harmonization.

### Harmonization Results

**Figure 1:**
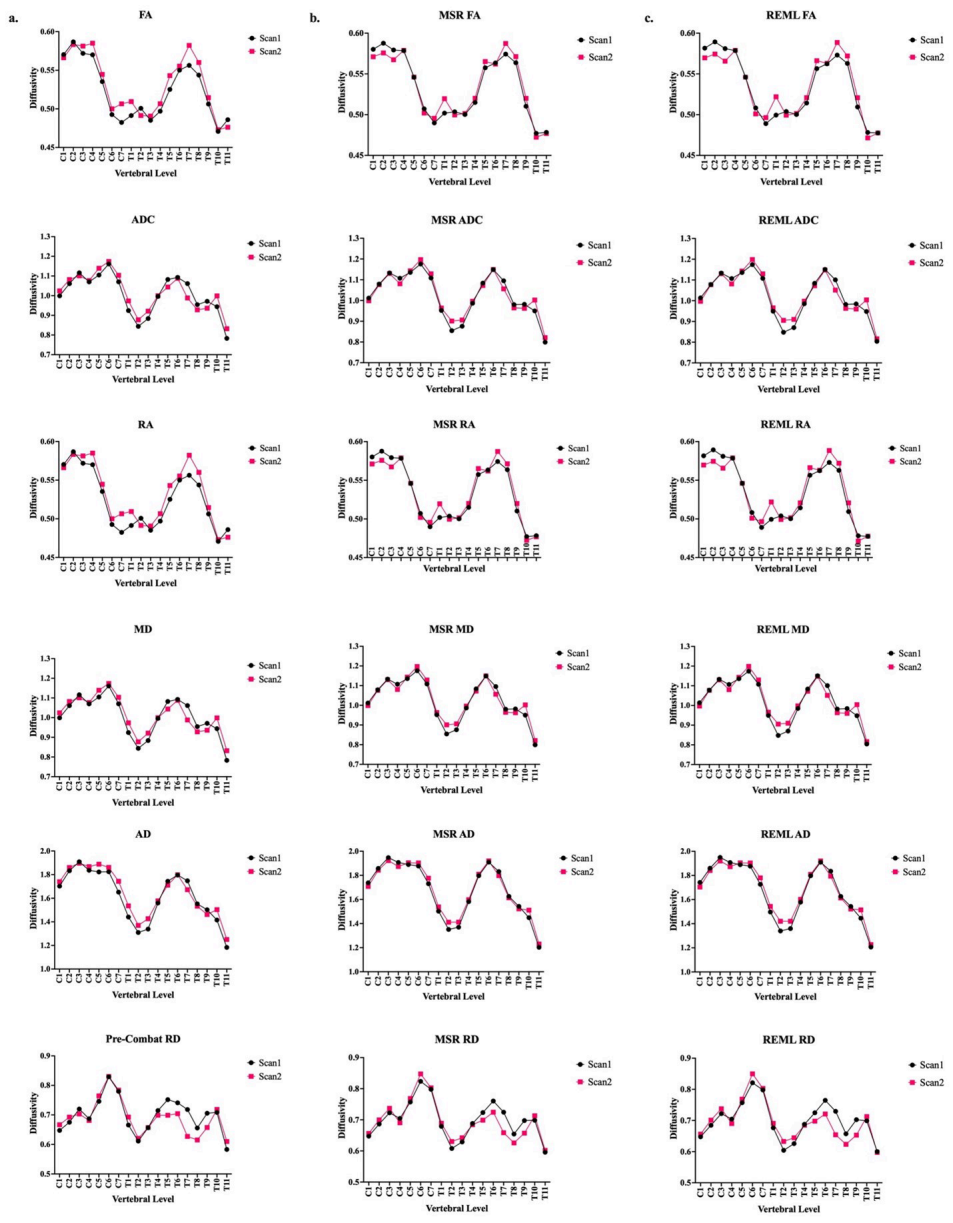
a) Pre-harmonized, averaged DTI metrics for each vertebral level for Scan 1 and Scan 2. b) Post-harmonized, averaged DTI metrics for each vertebral level for Scan 1 and Scan 2 using the MSR model. c) Post-harmonized, averaged DTI metrics for each vertebral level for Scan 1 and Scan 2 using the REML model.

The averaged values of FA, ADC, RA, MD, AD, and RD across subjects exhibited increased consistency between scans after harmonization with both MSR and REML model of longComBat (Fig. 1). Harmonization with both the MSR and the REML models led to enhanced correlation between longitudinal scans across all subjects, as evidenced by higher Pearson correlation values (Table 1). Harmonization with the MSR model showed slightly stronger correlation improvement as compared to the REML model.

There was minimal improvement in intra-subject agreement between scans when using the MSR model for harmonization, and intra-subject agreement decreased with the REML model (Table 2).

## Discussion

In our prior study, we have showed longComBat as a reliable tool to reduce scanner effects and improve agreement between datasets acquired from different scanners at multiple sites. In contrast, this study has shown that the harmonization approach with longComBat was not effective in improving agreement and reducing variance between longitudinal scans acquired from a single scanner. Averaged DTI metrics exhibited limited improvement in agreeability and consistency post-harmonization on an intrasubject level. The longComBat offers two models, REML and MSR, with REML being the default. Interestingly, REML did not perform well in harmonizing longitudinal data in this study and, in fact, could negatively impact the results for such data. Therefore, the longComBat with REML model should be used with caution in longitudinal data with limited scanner effects.

There should be no expectation that longComBat can significantly reduce the variability in single-scanner data. Given that diffusivity metrics are directly related to absolute attenuation between unweighted and diffusion weighted images, the results are expected since there are no differences in acquisition parameters. Therefore, the variability observed in longitudinal scans are most likely attributable to physiological motion, and longComBat was not able to successfully mitigate that effect. Average values for all metrics show substantial decreases in the upper thoracic region due to artifact from respiratory motion/cardiac pulsation. We have previously shown that differences in diffusion acquisition parameters including the presence/absence of gating can result in over or underestimation of diffusion magnitude in this region [14].

There have been few studies that showed good reproducibility of DTI cervical spinal cord imaging in healthy subjects within scanner [24, 25], but we believe this is the first study that examines the reproducibility of the cervical and thoracic spinal cord reproducibility in both healthy and spinal cord injury pediatric population within scanner. We have showed that there is relatively good agreeability between longitudinal scans in both cervical and thoracic spinal cord, but the utility of longComBat in improving the reproducibility is limited.

Spinal cord DTI has been increasingly studied as a potential source of biomarkers for pathology, but reproducibility has been hindered by scanner hardware differences, pulse sequence variabilities, physiological motions, and subject compliance concerns, particularly in clinical settings. Despite efforts in standardizing scanning protocols and procedures across sites, challenges remain in combining DTI datasets due to technical limitations or time constraints. The lack of reproducibility is a major limitation to combine DTI datasets from multiple sites and time points in research studies. In our studies, longComBat has consistently shown promising results to reduce additive and multiplicative scanner effects across sites, but its use is limited in reducing variability between longitudinal scans within scanner with short inter-scan interval, given limited scanner effects contributing to the variability. The effectiveness of longComBat in reducing variability between scans within scanner with longer longitudinal time frame, such as numerous months, cannot be predicted and remains a future research interest.

## Methods

### Subject Recruitment

Forty-two healthy pediatric volunteers and twenty-seven spinal cord injury pediatric volunteers were recruited for this study. Healthy subjects (n = 42) including 25 females and 17 males ranged in age from 6–16, with a mean age of 11.57, and spinal cord injury subjects (n = 27) including 13 females and 14 males ranged in age from 6–16, with a mean age of 11.64. All the healthy subjects had no history of spinal cord pathology or injury. Subjects with SCI were required to meet the following criteria: stable cervical or thoracic SCI, at least 6 months post-injury, and no metal instrumentation.

### Diffusion Imaging & Processing

All the Diffusion weighted images for DTI analysis were acquired using an inner-field-of-view EPI sequence to provide short scan time, reduced geometric distortion, and improved SNR [21]. Due to the small size of the spinal cord and need for high in-plane resolution this type of small field of view sequence is extremely valuable in collecting spinal cord DTI. DTI data was collected in 6 mm axial slices parallel to the spinal cord. Two slabs were acquired, one covering the cervical to upper/mid-thoracic, and a second covering the upper/mid-thoracic to the T12-L1 disc with a minimum of one level of vertebral overlap to ensure complete coverage. Imaging parameters for the DTI sequence were TE = 110 ms, TR = 7900 ms, diffusion weighted directions = 20, b = 800 s/mm^2^, acquisition voxel size = 0.8 × 0.8 × 6 mm^3^, axial slices = 40, averages = 3, b0 acquisitions = 6. Cardiac and respiratory gating were not used in order to keep scans as short as possible given the age of the participants. After acquisition, DTI data was motion corrected, and co-registered to the b0 image using a 3- D rigid body transformation in order to compensate for motion during the scan. Next, tensor estimation was performed using a non-linear fit implementation of the RESTORE robust outlier rejection algorithm to mitigate the impact of image artifact, noise, or misregistration and DTI metrics FA, MD, AD, and RD were generated from the calculated diffusion tensors [22].

DTI metrics were aggregated for all subjects based on acquisition parameters and vertebral levels for subsequent analysis. Mean values were computed for each vertebral level. Pearson correlations for average values per vertebral level were computed between scans using Prism software. Additionally, intra-class correlation coefficients (ICCs) were calculated to assess agreement per vertebral level using ICC2 [23] with the R software.

### Harmonization

Data harmonization was conducted for both the initial and repeated scan data utilizing the longComBat technique, MSR and REML methods [20]. Prior to the application of longComBat, all DTI metrics were organized by subject, scanner, and vertebral level. Each subject contributed data from two time points, with the scan before the two-hour break designated as timepoint one, and the scan after the break as timepoint two. Age and sex were included as covariates, while the remaining factors were grouped as scanner effects. Custom scripts written in Python and R were utilized to perform the harmonization process. Following harmonization, averaged data for all subjects was compared pre- and post-harmonization to assess changes in agreement. Additionally, ICC values and Pearson scores were examined both before and after harmonization.

## Figures and Tables

**Table 1:** Pearson correlation values between the two scans based on average value by vertebral level showing original, harmonized results using the MSR model, and harmonized results using the REML model.

	Original	MSR	REML
AD	0.97	0.99	0.99
ADC	0.94	0.98	0.97
FA	0.96	0.98	0.96
MD	0.94	0.98	0.97
RA	0.96	0.98	0.96
RD	0.85	0.92	0.89

**Table 2:** ICC values at 95% confidence interval for both longitudinal scans using the MSR and REML model, showing lower (LB) and upper (UB) bounds. Values were computed for all metrics by vertebral level.

	ICC (LB, UB)		
	Original	MSR	REML
AD	0.63 (0.57, 0.68)	0.65 (0.59, 0.70)	0.54 (0.48, 0.60)
ADC	0.53 (0.46, 0.59)	0.55 (0.48, 0.60)	0.45 (0.38, 0.52)
FA	0.67 (0.62, 0.72)	0.68 (0.63, 0.73)	0.54 (0.48, 0.60)
MD	0.53 (0.46, 0.59)	0.55 (0.48, 0.60)	0.45 (0.38, 0.52)
RA	0.67 (0.62, 0.72)	0.68 (0.63, 0.73)	0.54 (0.48, 0.60)
RD	0.50 (0.43, 0.56)	0.51 (0.44, 0.57)	0.43 (0.35, 0.50)

## Data Availability

The datasets generated and analyzed during the current study are available from the corresponding author on reasonable request.
